# External Validation and Retraining of DeepBleed: The First Open-Source 3D Deep Learning Network for the Segmentation of Spontaneous Intracerebral and Intraventricular Hemorrhage

**DOI:** 10.3390/jcm12124005

**Published:** 2023-06-12

**Authors:** Haoyin Cao, Andrea Morotti, Federico Mazzacane, Dmitriy Desser, Frieder Schlunk, Christopher Güttler, Helge Kniep, Tobias Penzkofer, Jens Fiehler, Uta Hanning, Andrea Dell’Orco, Jawed Nawabi

**Affiliations:** 1 Department of Radiology, Charité—Universitätsmedizin Berlin, Freie Universität Berlin, Humboldt-Universität zu Berlin, Charitéplatz 1, 10117 Berlin, Germany; 2 Neurology Unit, Department of Neurological Sciences and Vision, ASST-Spedali Civili, 25123 Brescia, Italy; 3 Department of Brain and Behavioral Sciences, University of Pavia, 27100 Pavia, Italy; 4 U.C. Malattie Cerebrovascolari e Stroke Unit, IRCCS Fondazione Mondino, 27100 Pavia, Italy; 5 Department of Neuroradiology, Charité School of Medicine and University Hospital Berlin, 10117 Berlin, Germany; 6 Department of Diagnostic and Interventional Neuroradiology, University Medical Center Hamburg Eppendorf, 20246 Hamburg, Germany; 7 Berlin Institute of Health (BIH), BIH Biomedical Innovation Academy, 10178 Berlin, Germany

**Keywords:** intracerebral hemorrhage, automated segmentation, deep learning, multicenter, external validation

## Abstract

Background: The objective of this study was to assess the performance of the first publicly available automated 3D segmentation for spontaneous intracerebral hemorrhage (ICH) based on a 3D neural network before and after retraining. Methods: We performed an independent validation of this model using a multicenter retrospective cohort. Performance metrics were evaluated using the dice score (DSC), sensitivity, and positive predictive values (PPV). We retrained the original model (OM) and assessed the performance via an external validation design. A multivariate linear regression model was used to identify independent variables associated with the model’s performance. Agreements in volumetric measurements and segmentation were evaluated using Pearson’s correlation coefficients (r) and intraclass correlation coefficients (ICC), respectively. With 1040 patients, the OM had a median DSC, sensitivity, and PPV of 0.84, 0.79, and 0.93, compared to thoseo f 0.83, 0.80, and 0.91 in the retrained model (RM). However, the median DSC for infratentorial ICH was relatively low and improved significantly after retraining, at *p* < 0.001. ICH volume and location were significantly associated with the DSC, at *p* < 0.05. The agreement between volumetric measurements (r > 0.90, *p* > 0.05) and segmentations (ICC ≥ 0.9, *p* < 0.001) was excellent. Conclusion: The model demonstrated good generalization in an external validation cohort. Location-specific variances improved significantly after retraining. External validation and retraining are important steps to consider before applying deep learning models in new clinical settings.

## 1. Introduction

Spontaneous intracerebral hemorrhage (ICH) is a major cause of morbidity and mortality worldwide despite the relatively small contribution to all stroke types of up to 27% [1,2,3]. The prognosis after ICH is particularly affected by the ICH volume in addition to its location, the presence of intraventricular hemorrhage (IVH), and acute hematoma expansion (HE) [4]. Thus, instruments for accurate ICH and IVH quantification upon neuroimaging are crucial to guide further patient management and to inform future clinic trials [5,6,7,8]. The ABC/2 method has remained a clinically well-established formula to manually estimate the ICH volume [9] despite the consistent reports of underestimation or overestimation in large and irregular bleedings. The semiautomatic measurements of ICH and IVH are equally limited as they are labor-intensive and time-consuming [10]. Novel deep learning-based models have the potential to quantify ICH and IVH volumes rapidly and accurately in a fully automated approach and are therefore in high demand [11]. The DeepBleed network presented by Sharrock et al. is the first publicly available 3D neural network for the segmentation of ICH and IVH [12]. Despite being trained and internally validated on a large dataset from the MISTIE II and III trial series [13,14], its performance in an independent cohort has not been described yet. This step is of particular importance in imaging-based segmentation networks as they have increasingly shown inconsistent performance results on external datasets [15]. Therefore, it is important that clinicians are aware of the quality assessment steps that need to be taken before the local implementation of these models. In particular, the performance of DeepBleed in the detection and segmentation of infratentorial and small ICH remains undetermined as these two subsets were excluded from the MISTIE trials [13,14,16]. The aim of this study was to evaluate the generalizability and further improve the robustness of the proposed DeepBleed network. The objective of this study was to assess the performance of the existing DeepBleed network before and after retraining. Therefore, we hypothesized that the DeepBleed network would accurately detect and segment ICH and IVH regardless of its location and size. To test and evaluate this, the following threefold steps were performed. First, we externally validated the original DeepBleed model (OM) in an independent multicenter cohort. Secondly, we retrained the model (RM) to test the effect on the validation accuracy through an internal validation design. Third, we compared the interrater reliabilities between the OM and RM network and independent human raters. This study serves as a use case illustrating how the generalizability of deep learning models may be addressed via local retraining.

## 2. Materials and Methods

### 2.1. Study Population

**Figure 1:**
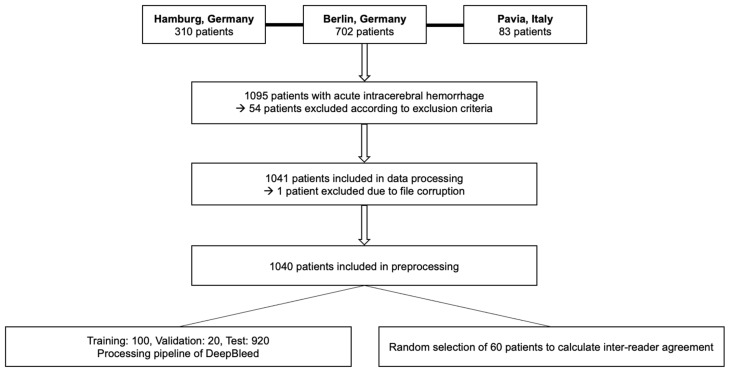
Flow diagram of patients included from three European participating sites and final patient cohort after further exclusion.

This retrospective study was approved by the local ethics committee (Charité Berlin, Germany (protocol number EA1/035/20), University Medical-Center Hamburg, Germany (protocol number WF-054/19), and IRCCS Mondino Foundation, Pavia, Italy (protocol number 20190099462]). Written informed consent was waived by the institutional review boards. All study protocols and procedures were conducted in accordance with the Declaration of Helsinki. The study included patients of ≥18 years who were diagnosed with primary spontaneous ICH upon noncontrast computed tomography (NECT) between January 2017 and June 2020. Patients with multiple ICH, artifacts, external ventricular drain (EVD) or any other type of surgical procedure, and secondary hemorrhage following head trauma, ischemic infarction, neoplastic mass lesions, ruptured cerebral aneurysms, or vascular malformations were excluded from the study as presented in Figure 1.

### 2.2. Image Acquisition and Manual Segmentation

Participating sites acquired NECT images according to their local imaging protocols. De-identified and pseudomyzed imaging data were retrieved from the local picture archiving and communication system (PACS) servers and converted into a Digital Imaging and Communications in Medicine (DICOM) format according to local guidelines. DICOM data were then transformed in Neuroimaging Informatics Technology Initiative (NifTI) for further imaging analysis. Images were analyzed for the presence of IVH and the ICH location by one experienced neuroradiologist (J.N., who has 5 years of experience in ICH imaging research). Supratentorial bleedings in cortical and subcortical locations were classified as lobar and hemorrhages involving the thalamus, basal ganglia, internal capsule and deep periventricular white matter [17]. Infratentorial bleedings were classified within the brainstem, pons and cerebellum [18]. Ground truth (GT) masks of ICH and IVH were manually segmented on CT scans by two experienced raters (both with 3 and 5 years of experience in ICH imaging research) who were supervised by one neuroradiologist (J.N.) who inspected each ICH and IVH mask for the quality of segmentations and corrected them if necessary. Segmentation of the ICH and IVH was performed using ITK-SNAP software version 3.8.0 (Penn Image Computing and Science Laboratory, Philadelphia, PA, USA) [19]. Two expert raters segmented the test set six months apart for 60 patients to calculate inter-reader agreement (J.N. and FM, with 5 years of experience in ICH imaging research). The function shuffle from Python library NumPy random was applied on the subject ID list [20]. All readers independently analyzed and segmented images in a random order while blind to all demographic data and were not involved in the clinical care of assessment of the enrolled patients.

### 2.3. Preprocessing and Postprocessing

**Figure 2:**
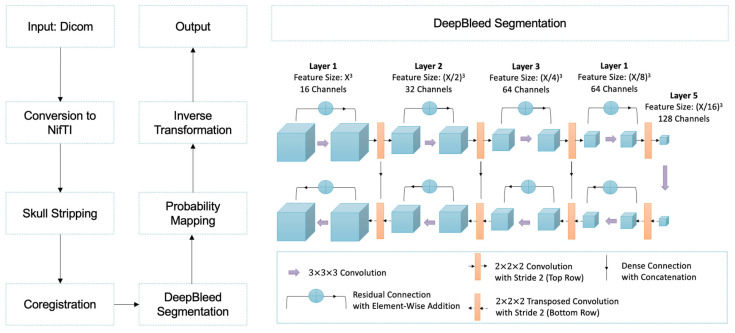
Processing pipeline of the 3D DeepBleed network.

The preprocessing comprised two steps as described in the original study (Figure 2): brain extraction and the coregistration. Briefly, after setting to zero the CT scan intensities lower than 0 or higher than 100, brain extraction was performed with FSL Brain Extraction Tool (BET) [21], setting the fractional intensity parameter (-f flag) to 0.01. Rigid coregistration was performed with ANTs [22] using a 1.5 mm^3^ isotropic CT template [23]. The resulting transformation was applied to the GT masks as well. During postprocessing, a threshold of 0.6 was applied to the resulting probability maps, setting the higher values to 1 and the others to 0. Finally, inverse coregistration of the resulting mask was performed using the inverse transformation matrix.

After gantry tilt and unequal slices were corrected, DICOM data were converted using NIfTI. For brain extraction and coregistration, Python preprocessing pipelines were used. DeepBleed was then used to predict intracerebral hemorrhage (ICH) and intraventricular hemorrhage (IVH). In the final step, the predictions from the previous template registration were inversely transformed in the native space.

### 2.4. Model Retraining

In addition to the general exclusion criteria described in the above, the following additional exclusion criteria were applied to the training cohort in accordance with those of MISTIE studies [13,14]: symptom onset > 24 h prior to the admission CT or an unknown time of symptom onset as well as an admission ICH volume of >30 mL. As described in the original study, adaptive moment estimation (Adam) was used as the optimization function [24], the dice similarity coefficient (DSC) was used as a loss function [25], and 100, randomly selected subjects were used as a training cohort, whereas 20 were used as a validation cohort. After testing various combinations, the optimal learning rate was 1 × 10^−4^ and a batch size of 3 was chosen. Initially, the training dataset was shuffled; we stopped the training if, after the first 10th epoch, the current epoch did not improve, and validation was performed every five epochs.

### 2.5. Model Testing

For the model testing, preprocessing and postprocessing as described above were performed on the OM and RM test dataset. Contrary to the training dataset, the test dataset was defined according to the original criteria of our study with no additional exclusion criteria applied. This decision was made because we were interested in evaluating the performance of DeepBleed on small hemorrhages.

### 2.6. Code Availability

The code is publicly available in Jupiter Notebooks on GitHub (Microsoft, San Fransisco, CA, USA) with the following link: https://github.com/Orangepepermint/retraindeepbleed accessed on 9 May 2023. It is written in Python v3.9 [26] using the following libraries: NumPy v1.23.3 [20], FSLpy v3.9.0 [27], and ANTsPy v0.3.1 [22].

### 2.7. Statistical Analysis

Statistics were conducted using GraphPad Prism v9.0.2 (GraphPad Software, Inc., San Diego, CA, USA) [28] and R (the R project for statistical computing, Vienna, AT) using tidyverse [29]. Various metrics were used to evaluate the DeepBleed performance and compare the segmentations from our RM with the OM. These included DSC, sensitivity, positive predictive value (PPV), and volume measurements. *t*-tests were used to compare the DSC, sensitivity, and PPV distributions between the OM and RM. Based on the central limit theorem, the *t*-test assumptions were fulfilled. To determine factors influencing segmentation performance, a linear regression model with the following formula was used:

DSC ~ volume+hemorrhage location+IVH presence+participating center

Pairwise correlations among volumes measured from each of the three segmentation methods (GT masks, OM and RM DeepBleed network) were assessed using the Pearson correlation coefficient (r). Agreements between two raters and the OM and RM DeepBleed network were assessed using the intraclass correlation coefficient (ICC) in the DSC. Moreover, a repeated measures ANOVA was performed with a pairwise *t*-test as a post hoc. The homogeneity of variance assumption for ANOVA was evaluated using the Levene test. Cohen’s d effect size was determined as well. A *p*-value of < 0.05 was considered significant. Bonferroni adjustment was applied where necessary. Adjusted *p*-values are indicated as *p*_adj_-values.

## 3. Results

### 3.1. Demographics and Characteristics of the Study Cohort

The manual review of the images led to an elimination of 54 patients due to exclusion criteria and manual segmentation errors. The final dataset was composed of 1040 patients, NECT scans and respective masks, where the numbers of patients in training, validation and test were *n* = 100, *n* = 20 and *n* = 920. The mean age was 69.6 (SD 14.2) years. The median NIHSS and GCS scores were 7.5 (IQR 12) and 13 (IQR 7), respectively. Imaging was performed within a median symptom onset time of 4.3 h (IQR 13.6). In total, 519 patients (49.9%) presented with ICH and IVH with a mean volume of 74.9 (SD 41.6) ml. A total of 521 patients (50.1%) presented with ICH only with a mean volume of 41.5 (SD 32.8) mL. No significant differences in demographic characteristics were found between training, validation, and test subjects as presented in Table 1.

### 3.2. Model Retraining and Testing

**Figure 3:**
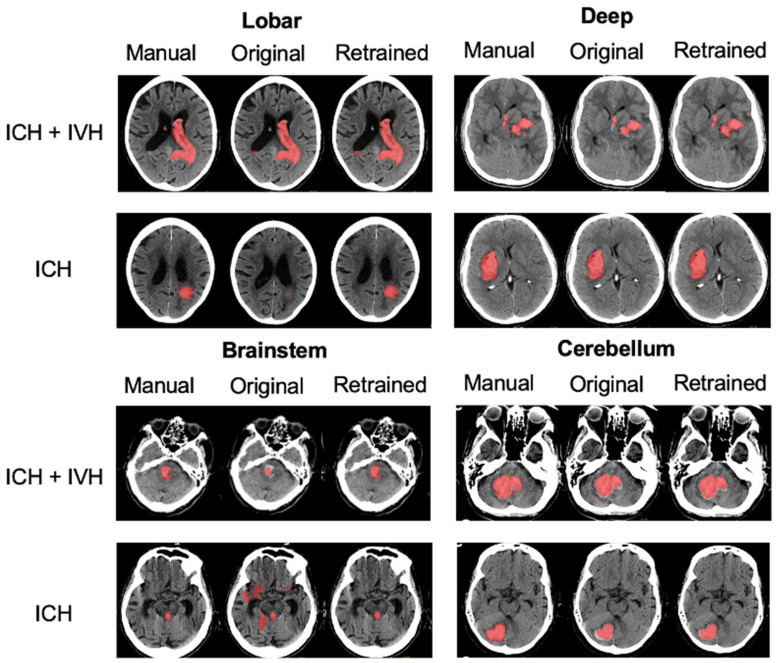
Segmentations of the original and retrained model across different locations. Illustrative examples of ground truth segmentations (red) for intracerebral hemorrhage (ICH) with intraventricular hemorrhage (IVH; upper rows) and ICH only (lower rows) given for deep, lobar, brainstem, and cerebellar ICH. Segmentations given are displayed for manual segmentations (left column), and DeepBleed segmentations with the original model (OM, middle column), and the retrained model (RM, right column).

**Figure 4:**
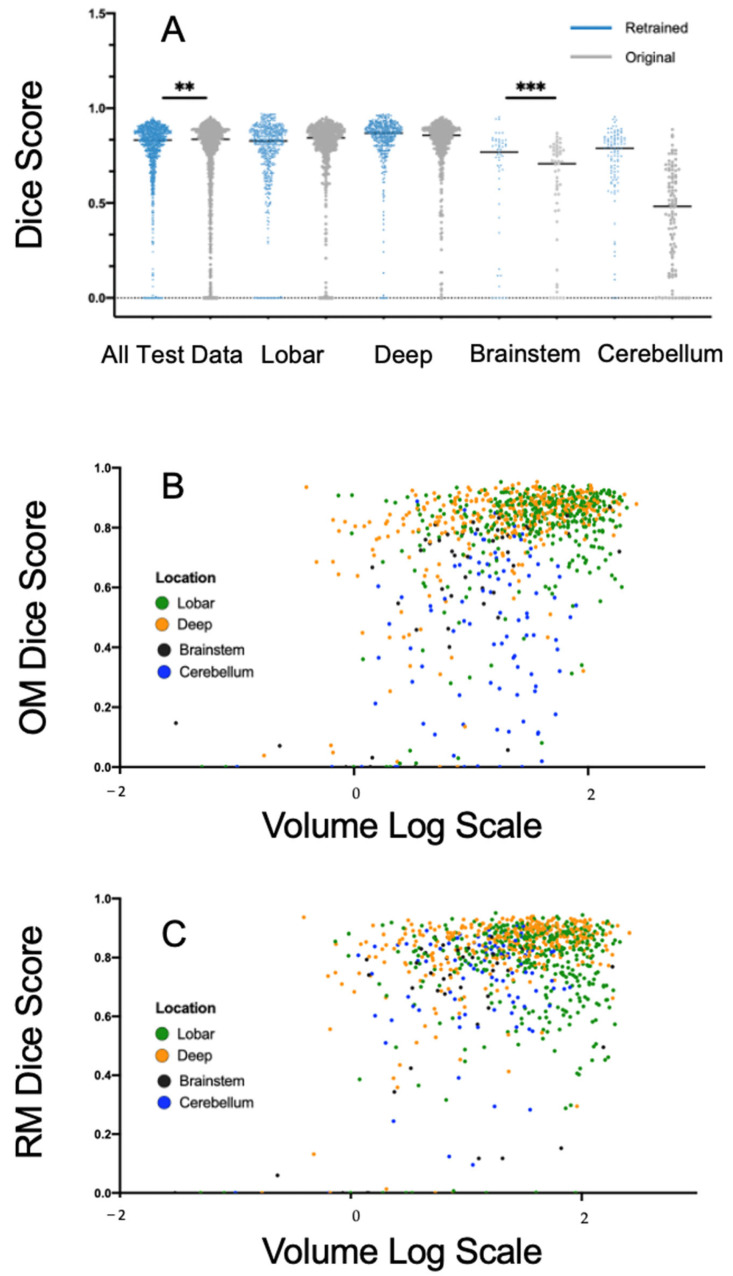
Model performance of the original and retrained model across different locations. (**A**) Comparison of dice scores across lobar, deep, brainstem and cerebellar hemorrhage with original weights (OM, grey) and retrained weights (RM, blue). (**B**,**C**) Relationship between hemorrhage volume and dice scores across different locations. (**B**) Automatic segmentation with OM. (**C**) Automatic segmentation with RM. **: *p*-value < 0.01, ***: *p*-value < 0.001.

With Nvidia RTX 3090 GPU, the model was trained for 810 epochs in 16 h. Illustrative examples of segmentations are displayed in Figure 3. Model performance metrics of the OM and RM derived in the test set are presented in detail in Table 2 with additional DSC metrics illustrated in Figure 4A. The overall DSC values for the segmentations across all locations were relatively similar in both DeepBleed models with a median DSC of 0.84 (95% CI, 0.73–0.88) in the OM and 0.83 (95% CI, 0.74–0.88) in the RM. DSC values given separately for each location were also almost equally high in supratentorial ICH with a median DSC of 0.86 (95% CI, 0.80–0.89) in deep ICH and 0.84 (95% CI, 0.78–0.89) in lobar ICH in the OM compared to 0.87 (95% CI, 0.81–0.90) and 0.83 (95% CI, 0.72–0.88) in the RM, respectively. In comparison, performance metrics in infratentorial locations demonstrated an overall lower DSC with a median DSC of 0.71 (95% CI, 0.46–0.78) in cerebellar ICH and 0.48 (95% CI, 0.23–0.64) in brainstem ICH in the OM. DSC metrics improved in the RM, especially in cerebellar ICH, with a median DSC of 0.79 (95% CI, 0.65–0.84) and 0.77 (95% CI, 0.57–0.83) in brainstem ICH. OM and RM performance metrics were significantly different for the DSC and PPV, at *p*_adj_-values of < 0.001, compared to the sensitivity, *p*_adj_-value of >0.05. DSC, and sensitivity showed a significant improvement in the cerebellum (*p* < 0.001) after retraining.

### 3.3. Analysis of Factors Influencing the Model Performance

The results from the multivariate linear models are summarized in Table 3. Results for the univariate analysis are presented in the Supplementary Table S2. Overall model performance was negatively influenced by the ICH location. However, deep ICH was the only location that was not significantly associated with a DSC loss performance neither in the OM nor RM network, at *p*-values of > 0.05. While the slope coefficients for lobar ICH remained relatively similar in the OM and RM (−0.04, SD 0.01 and −0.06, SD 0.01), the negative effect of brainstem and cerebellar ICH decreased from −0.20 (SD 0.03) and −0.32 (SD 0.02) in the OM to −0.18 (SD 0.03) and −0.08 (SD 0.02) in the RM, at *p*-values of < 0.001. ICH volume increase had a strong positive effect on the DSC in the OM and RM network. The presence of IVH and the data’s originating site had no significant effect on the DSC in the OM or RM. The correlation between ICH location and volume in the DSC are illustrated in Figure 4B,C.

### 3.4. Volume and Segmentation Agreement Analysis

**Figure 5:**
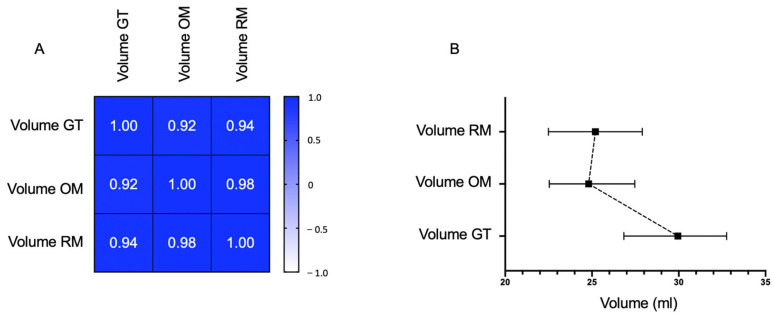
Volume agreement analysis of the original and retrained model with the ground truth. (**A**) Pearson’s correlation matrix of agreement for ground truth volume and automatic segmentations of DeepBleed with original weights (original model, OM) and retrained weights (retrained model, RM). (**B**) Median intracerebral hemorrhage and intraventricular hemorrhage volumes of ground truth and automatic segmentation with OM and RM given with a 95% confidence interval. The mean volumes of GT, OM and RM (± SD) were 43.2 (± 42.6), 36.0 (± 35.9) and 36.2 (± 35.9). It is possible to see that DeepBleed normally underestimates volume, probably due to the probability map threshold.

Figure 5A shows the correlation between the GT masks and DeepBleed’s automatic volume prediction with the OM and RM. Overall strong correlations were observed among the three segmentation methods (r > 0.9, *p*-value < 0.001), however, correlations among both DeepBleed models, OM and RM, were highest, whereas their correlation with GT volumes was lowest (r = 0.92 for OM and r = 0.94 for RM). The mean volumes of GT, OM and RM (±SD) were 43.2 (±42.6), 36.0 (±35.9) and 36.2 (±35.9). The median volumes of the three methods are displayed in Figure 5B. The repeated-measure ANOVA showed no significant effect between the volume estimation of GT masks and automatic volume estimations in the DeepBleed OM and RM (F = 0.45, *p*-value > 0.05).

**Figure 6:**
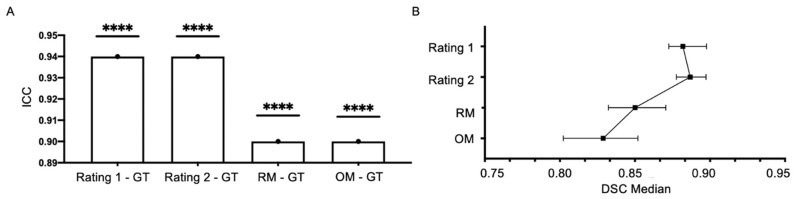
Segmentation agreement analysis of the original and retrained model and human expert raters. (**A**) Intraclass correlation coefficient (ICC) of dice score (DSC) from two experienced raters, automatic segmentations of DeepBleed with original weights (OM) and retrained weights (RM) compared to ground truth (GT) of a senior stroke imaging neuroradiologist. (**B**) Median with 95% CI of DSC from each group. DSC, dice score; ICC, intraclass correlation coefficient; OM, original model; RM, retrained model. ****: *p*-value < 0.0001.

Significant agreements were found between the DSCs of manual segmentations by two expert raters and those of the OM and RM DeepBleed network with the GT masks (ICC = 0.90 and ICC = 0.94, *p*-value < 0.0001) and are presented in Figure 6A,B. The repeated-measure ANOVA showed a significant effect of the rater and OM and RM on the DSC (F = 14.38, *p* < 0.0001). The post hoc test showed a significant effect between OM and RM (t = 2.78, *p*_adj_-value < 0.05, d = 0.4, small), OM and both raters (t = −5.11 and t = –5.37, *p*_adj_-value <0.001 and d = 0.7, moderate for both) and RM and both raters (t = −4.9 and t = −5.3, *p*_adj_-value < 0.001 and d = 0.7, moderate for both). No significant difference was found between the two raters (t = −1.57, *p*-value > 0.05, d = 0.2, small).

## 4. Discussion

Our study confirmed the external validity of the first open-source 3D deep learning network for the automatic detection and segmentation of spontaneous ICH with the presence of IVH upon CT. Furthermore, we illustrated the importance of local retraining for a specific setting to increase the applicability of neural network models for ICH segmentation purposes.

In brief, the OM showed overall good results in our multicenter cohort during the validation process. However, location-specific performance metrics were comparatively low for infratentorial ICH lesions which improved significantly after retraining. In particular, performance metrics in cerebellar ICH improved with a 1.7-fold increase in the DSC. The negative effects on the DSC in our linear model improved with a slope increase of 25% for cerebellar ICH after retraining. In comparison, the model performance in supratentorial ICH was overall good for both the OM and RM, with deep ICH demonstrating the best and stable performance metrics. Nonetheless, even lobar ICH slightly improved after model retraining while initially demonstrating the second-best performance metrics in the OM. Additionally, the DSC performance was negatively associated with lobar ICH lesions in our linear regression model. Lobar ICH may demonstrate irregular margins and internal density heterogeneities upon imaging [30]. These imaging phenomena have especially been associated with the use of oral anticoagulants which in turn have been excluded from the MISTIE III trial (novel oral anticoagulants; NOAC) [14,31,32]. As DeepBleed adopts a binary prediction approach at the voxel level using a predetermined threshold, these nuanced voxel differences might be missed [12]. In comparison, another deep learning network, nnU-Net, utilizes the softmax output, as demonstrated by Zhao et al., for ICH segmentation [33,34,35]. However, we believe that these differences have only a minor impact on the general segmentation performance as shown by the overall good DSC during the validation process. A key strength of the network was that the DSC metrics were independent of the participating site’s dataset as well as the presence of IVH. The level of heterogeneity in the developmental cohort of the DeepBleed network may directly relate to this high generalizability of the original model in our external validation cohort. In brief, the original DeepBleed network was multicenter-curated with data from the MISTIE II and III trial series that were conducted at 78 sites in North America, Europe, Australia, and Asia with over 500 patients included [13,14]. In comparison, most of the previous ICH segmentation models were single-center-curated and thus required even more generalization testing ahead of clinical implementation at other sites [35,36,37,38,39]. Secondly, the DeepBleed model employs a dice-based loss function and Adam optimizer, enabling the easy combination of various datasets and augmentation options making it easy to share the trained models as open-source models in order to adapt the network to specific settings [12].

We also observed some limitations in the DeepBleed network. We found that a volume increase had a significantly positive effect on the DSC. This finding is also consistent with that of previous studies showing a positive correlation between lesion size and the DSC in other segmentation networks [40,41]. Therefore, DeepBleed’s limited performance in small ICH appears to be a general limitation of deep learning-based networks for segmentation purposes rather than a methodological limitation of DeepBleed—due to the inclusion of supratentorial ICH with an absolute volume greater 30 mL according to the inclusion criteria of the MISTIE III trial [14]. Considering other performance metrics, the absolute volume differences described by the DeepBleed authors were only small despite the variations in the DSC [12] and were thereby within the range with volume errors of 2 to 5 mL observed in similar studies [11,12,42]. In line with this, our post hoc analysis showed a high correlation between the manual and automatic volume predictions, with an underestimation of 5 mL in the automated approach, while the automatic segmentations had a statistically lower agreement in terms of the DSC compared to human expert raters [43,44]. The latter findings might have a stronger clinical implication than do the conclusions obtained from the DSC metrics, as in clinical practice the ICH volume is of great relevance [13,14,16,45,46,47]. The DSC evaluates the quality of the alignment, which denotes the overlap between the predicted and the GT segmentation [48]. Finally, our results are limited to an external validation cohort of ICH patients who presented within a symptom onset of 24 h. Hence, the performance upon follow-up CT scans beyond a time interval of 24 h may differ.

Our study has the following two main implications. First, our results illustrated that the generalizability of neural networks may be restricted, even when the development and validation cohorts have strong similarities in terms of patient population and healthcare context. Secondly, more extensive retraining may be required to improve the performance at a new site when generalizability is poor. From a clinical point of view, the open-source RM could support decision making for surgical interventions and aid outcome prediction [18,49] in both supra- and infra-tentorial ICH [17,18] with IVH [50,51].

To conclude, the DeepBleed network demonstrated good generalization in an external validation cohort of patients diagnosed with spontaneous ICH on CT and retraining improved location-specific variances significantly. Volumetric analysis showed strong agreement with the manual segmentations of expert raters. However, segmentation accuracy was statistically higher in ground truth masks. The code and RM weights have been made available online [52,53]. Our study illustrates the importance of local retraining for a specific setting to increase the applicability of neural network models for segmentation purposes in spontaneous ICH patients. ICH clinicians and decision makers may take this into account when considering applying externally designed neural network models to their local settings.

## Figures and Tables

**Table 1:** Comparison of the demographics and volumetry.

Variable		Training Cohort (*n* = 100)	Validation Cohort (*n* = 20)	Test Cohort (*n* = 920)	*p*-Value
Age (years), mean ± SD		70.5 ± 13.1	68 ± 13.4	69.6 ± 14.2	0.84 ^1^
Sex, *n* (%)					
	Male	41 (41)	11 (55)	516 (56)	
	Female	59 (59)	9 (45)	404 (44)	
NIHSS score, median (IQR)		7.5 (10)	10 (10)	7 (5)	0.70 ^1^
GCS score, median (IQR)		13 (7)	14 (4)	13 (8)	0.11 ^2^
Symptom onset to imaging (hours), median (IQR)		4.3 (13.6)	3.9 (7.6)	4.23 (12.8)	0.89 ^2^
ICH location, *n* (%)					
	Lobar	44 (44)	6 (30)	338 (36.7)	
	Deep	46 (46)	7 (35)	455 (49.5)	
	Brainstem	3 (3)	3 (15)	41 (4.5)	
	Cerebellum	7 (7)	4 (20)	86 (9.3)	
ICH + IVH volume (ml), mean ± SD		83.8 ± 47.2	56.4 ± 89.7	76.5 ± 44.9	0.67 ^2^
ICH volume (ml), mean ± SD		27.7 ± 30.2	34.2 ± 30.9	44.1 ± 14.2	0.30 ^2^
IVH volume (ml), mean ± SD		61.1 ± 49.1	22.2 ± 77.1	34.5 ± 42.5	0.64 ^2^

Demographics and descriptive characteristics compared between the training, validation, and test datasets. NIHSS, National Institutes of Health Stroke score; GCS, Glasgow Coma Scale; ICH, intracerebral hemorrhage; IQR, interquartile range; IVH, intraventricular hemorrhage; SD, standard deviation. ^1^ one-way-ANOVA test; ^2^ Kruskal–Wallis test, if the data do not fulfill the normal distribution.

**Table 2:** Original and retrained model performance metrics.

Metric	All Locations	Deep	Lobar	Brainstem	Cerebellum
OM					
DSC	0.84 (0.73, 0.88)	0.86 (0.80, 0.89)	0.84 (0.78, 0.89)	0.71 (0.46, 0.78)	0.48 (0.23, 0.64)
Sensitivity	0.79 (0.65, 0.86)	0.85 (0.79, 0.91)	0.80, (0.70, 0.87)	0.58 (0.38, 0.74)	0.34 (0.13, 0.49)
PPV	0.93 (0.85, 0.97)	0.91 (0.85, 0.95)	0.99 (0.85, 0.97)	0.88 (0.76, 0.94)	0.94 (0.76, 0.99)
RM					
DSC	0.83 (0.74, 0.88)	0.87 (0.81, 0.90)	0.83 (0.72, 0.88)	0.77 (0.57, 0.83)	0.79 (0.65, 0.84)
Sensitivity	0.80 (0.69, 0.87)	0.85 (0.79, 0.91)	0.79 (0.63, 0.88)	0.72 (0.57, 0.79)	0.75 (0.59, 0.84)
PPV	0.91 (0.84, 0.95)	0.91 (0.85, 0.95)	0.92 (0.63, 0.88)	0.87 (0.77, 0.94)	0.88 (0.79, 0.94)
t^1^ OM vs. RM (*p*_adj_-value)					
DSC	−5.9 (0.001)	1.64 (ns)	4.57 (0.001)	1.90 (ns)	12.94 (0.001)
Sensitivity	1.45 (ns)	3.05 (0.036)	4.03 (0.001)	3.33 (0.03)	16.49 (0.001)
PPV	−7.23 (0.001)	0.12 (ns)	2.33 (ns)	0.02 (ns)	0.30 (ns)

Model performance across the original (OM) and retrained (RM) DeepBleed models. All datasets and hemorrhage locations were evaluated for dice scores (DSCs), sensitivity, and positive predictive values (PPVs) and are given as medians with 95% confidence intervals. The metrics of the original (OM) and retrained weights (RM) were compared using *t*-tests with adjusted *p*-values. Briefly, 95% CI = 95% confidence interval; *p*_adj_-value = adjusted *p*-value; t^1^ = paired *t*-test between OM and RM for the specified metric; ns = not significant.

**Table 3:** Factors influencing the original and retrained model performance.

OM				RM		
Parameter	Slope	SD	*p*-Value	Slope	SD	*p*-Value
	0.75	0.01	<0.001	0.78	0.01	<0.001
Location (in respect to deep location)						
Lobar	−0.04	0.01	<0.01	−0.06	0.01	<0.001
Brainstem	−0.20	0.03	<0.001	−0.18	0.03	<0.001
Cerebellum	−0.32	0.02	<0.001	−0.08	0.02	<0.001
Volume (mm^3^)	0.00	0.00	<0.001	0.00	0.00	<0.001
IVH Presence	0.02	0.01	0.17	0.02	0.01	0.15
Center (in respect to Berlin, DE)						
Hamburg, DE	0.003	0.01	0.81	−0.02	0.013	0.09
Pavia, IT	0.008	0.02	0.73	−0.01	0.023	0.66

Multivariate linear regression analysis of variables influencing the model performance in the original (OM) and retrained (RM) DeepBleed model. DE, Germany; IT, Italy; IVH, intraventricular hemorrhage; SD, standard deviation.

## Data Availability

The data that support the findings of this study are available from the corresponding authors upon reasonable request and in accordance with the institution’s data security regulations.

## Supplementary Materials

The following supporting information can be downloaded at: https://www.mdpi.com/article/10.3390/jcm12124005/s1; Table S1: Original and retrained model performance metrics; Table S2: Factors influencing the original and retrained model performance using univariate analysis.
