# Supplementary material for: External Validation and Retraining of DeepBleed: The First Open-Source 3D Deep Learning Network for the Segmentation of Spontaneous Intracerebral and Intraventricular Hemorrhage
Source: J Clin Med. 2023 Jun 12;12(12):4005. doi: 10.3390/jcm12124005 (PMC10299035; doi:10.3390/jcm12124005)
Supplement: Supplementary file 1 [file jcm-12-04005-s001.zip › jcm-2417250-supplementary.pdf]

Supplementary Materials: Effect analysis of factors influencing the Dice Score

ANCOVA analysis on the OM showed that hemorrhage volume had an effect on the DSC (F=39.8, p<0.0001), PPV (F=4.8, p<0.0001) and sensitivity (F=4.6, p<0.0001). Only the DSC was significantly influenced by location (F=39.9, p<0.0001). There was no significant effect of IVH presence on metrics, while database had a significant effect on PPV (F=6.8, p<0.001).

The same statistical test on the RM showed a significant effect of hemorrhage volume on DSC (F=12.3, p<0.001) and PPV (F=29.5, p<0.0001) but not on sensitivity. DSC (F=14.3, p<0.0001), sensitivity (F=20.0, p<0.0001), but not PPV, were significantly affected by hemorrhage location. Furthermore, IVH presence had no effect on any metric, while the participating center only affected PPV (F=19.1, p<0.05).

Supplementary Table S1. Original and retrained model performance metrics.

Metric	All locations	Lobar	Deep	Brainstem	Cerebellar
**OM**					
DSC	75 ± 22	79 ± 19	81 ± 16	58 ± 28	43 ± 25
Sensitivity	71 ± 24	75 ± 21	79 ± 16	52 ± 26	33 ± 23
PPV	86 ± 19	87 ± 18	87 c 17	77 ± 29	82 ± 18
**RM**					
Dice Score	77 ± 19	76 ± 21	83 ± 16	64 ± 30	72 ± 20
Sensitivity	74 ± 20	73 ± 21	82 ± 15	64 ± 26	69 ± 23
PPV	86 ± 16	87 ± 15	87 ± 15	80 ± 23	82± 18

Model Performance across the original (OM) and retrained (RM) DeepBleed models. All datasets and hemorrhage locations were evaluated for dice scores (DSC), sensitivity, and positive predictive value (PPV) and are given as mean with standard deviation.

Supplementary Table S2: Factors influencing the original and retrained model performance using univariate analysis.

	OM			RM		
**Parameter**	**Slope**	**SD**	**p-value**	**Slope**	**SD**	**p-value**
**Location (in respect to deep location)**						
**Intercept**	0.81	0.01	<0.001	0.82	0.01	<0.001
**Lobar**	-0.02	0.01	0.09	-0.06	0.01	<0.001
**Brainstem**	-0.23	0.03	<0.001	-0.20	0.02	<0.001
**Cerebellum**	-0.37	0.02	<0.001	-0.11	0.02	<0.001
**Volume (mm^3)**						

**Intercept**	0.67	0.01	<0.001	0.73	0.01	<0.001
**Volume**	0.00	0.00	<0.001	0.00	0.00	<0.001
**IVH Presence**						
**Intercept**	0.71	0.01	<0.001	0.74	0.01	<0.001
**IVH Presence**	0.08	0.01	<0.001	0.06	0.01	<0.001
**Center (in respect to Berlin, DE)**						
**Intercept**	0.75	0.01	<0.001	0.77	0.01	<0.001
**Hamburg, DE**	0.02	0.02	0.27	-0.02	0.01	0.30
**Pavia, IT**	0.02	0.03	0.52	-0.01	0.02	0.65

Univariate linear regression analysis on variables influencing the model performance in the original (OM) and retrained (RM) DeepBleed model. DE, Germany; IT, Italy; IVH, intraventricular hemorrhage; SD, standard deviation.
